# Determination of Changes in Tissue Perfusion at Home with Hyperspectral and Thermal Imaging in the First Six Weeks after Endovascular Therapy in Patients with Peripheral Arterial Disease

**DOI:** 10.3390/diagnostics12102489

**Published:** 2022-10-14

**Authors:** Kirsten F. Ma, Thomas S. Nijboer, Simone F. Kleiss, Mostafa El Moumni, Reinoud P. H. Bokkers, Richte C. L. Schuurmann, Jean-Paul P. M. de Vries

**Affiliations:** 1 Division of Vascular Surgery, Department of Surgery, University of Groningen, University Medical Center Groningen, 9713 GZ Groningen, The Netherlands; 2 Division of Trauma Surgery, Department of Surgery, University of Groningen, University Medical Center Groningen, 9712 CP Groningen, The Netherlands; 3 Department of Radiology, Medical Imaging Center, University of Groningen, University Medical Center Groningen, 9712 CP Groningen, The Netherlands

**Keywords:** endovascular procedures, hyperspectral imaging, peripheral arterial disease, thermal imaging, tissue perfusion

## Abstract

The aims of this study were to assess changes in tissue perfusion up to 6 weeks after endovascular therapy (EVT), in hospital and at home, and to determine differences in tissue perfusion between patients with and without clinical improvement or good angiographic result. This single-center prospective cohort study included patients undergoing EVT for Rutherford stages two to six. Hyperspectral and thermal imaging were performed at the dorsal and plantar sides of the foot. These measurements consisted of a baseline measurement pre-EVT, and six follow-up measurements obtained at 1 and 4 h and 6 weeks in hospital, and 1 day, 7 days, and 14 days at home. Clinical improvement was defined as a decrease of one or more Rutherford class or decrease in the wound surface area and a good angiographic result was accomplished when a Transatlantic Inter-Society Consensus for the Management of PAD II C or D lesion was treated and uninterrupted flow continued in at least one below-the-knee artery in continuation with the inframalleolar arteries. The study included 34 patients with 41 treated limbs. Deoxyhemoglobin values were lower 1 h post-EVT compared with baseline and increased over time up to 6 weeks post-EVT. Significant differences in deoxyhemoglobin levels at 7 and 14 days post-EVT were determined between patients with and without clinical or angiographic success. This prospective pilot study shows the feasibility of hyperspectral imaging and thermal imaging post-EVT at home, which may decrease the need for hospital visits.

## 1. Introduction

With an aging population, there is an increase in multimorbid, fragile, and immobile patients with peripheral arterial disease (PAD) [1,2]. Endovascular therapy (EVT) is the preferred treatment to restore the blood flow in the lower extremity of PAD patients [1,3]. The technical success of EVT is currently determined by evaluating the patency of the main arteries on the completion angiography [4]. Recanalization, however, does not always lead to clinical improvement in a decrease in symptoms or wound healing in patients with chronic limb-threatening ischemia (CLTI) [3,5,6]. Patients are therefore closely monitored at the outpatient clinic post-EVT [7]. Besides clinical examination, imaging techniques, such as Doppler ultrasound, are often used to determine the patency of the arterial target lesions.

The actual improvement of tissue perfusion post-EVT is not regularly assessed, but seems crucial for predicting wound healing, decrease in ischemic pain, and to detect whether additional interventions or early reinterventions are needed [8,9,10,11,12]. PAD patients would benefit if these measurements could be performed at their homes. Lower-limb perfusion may gradually improve during the first weeks after the procedure, but previous studies are inconclusive about when improvement can be expected and the correlation with clinical outcomes [13,14]. Hyperspectral imaging (HSI) and thermal imaging are two techniques that may be suitable for monitoring tissue perfusion post-EVT, both in hospital and at home. Both techniques are non-invasive, contact-free, and are hand-held cameras that are easy to use in the home setting of patients [15,16,17].

The aim of this pilot study was to assess the changes in tissue perfusion, both in hospital and at home, up to 6 weeks post-EVT. The secondary aim was to determine whether there is an early detectable difference in tissue perfusion between patients with and without clinical improvement to predict outcomes at an early stage.

## 2. Materials and Methods

This single-center prospective cohort study included patients undergoing EVT for Rutherford classes 2 to 5. Patients were included between November 2019 and August 2021 at the University Medical Centre Groningen (UMCG). Patient inclusion was discontinued from March 2020 until August 2020 because of the coronavirus disease 2019 (COVID-19) pandemic. The UMCG Institutional Review Board approved the study protocol (METC #2019/114). Study procedures were performed according to the Medical Research Involving Human Subjects Act and the Declaration of Helsinki. The study was registered in the Netherlands Trial Register (#NL7713). Written informed consent was obtained from all patients.

### 2.1. Study Population

Patients with PAD scheduled for EVT were eligible for inclusion when the following criteria were met: age ≥ 18 years and PAD Rutherford stages 2 to 6 with indication for elective EVT. Exclusion criteria were illiteracy or language barrier, acute limb ischemia, severe peripheral edema, cellulitis, or erysipelas of the leg or foot. Patients were not excluded if the affected limb was previously treated. Baseline characteristics, including sex, age, body mass index, smoking status, comorbidities, Rutherford classification, and ankle-brachial index (ABI), were collected from medical records.

Of the 40 patients who participated in this study, 23 were previously included in a study investigating periprocedural changes with HSI and thermal imaging; however, that study focused on measurements directly before and after percutaneous transluminal angioplasty (PTA) [18].

### 2.2. Endovascular Revascularization

Indication for treatment was determined by a multidisciplinary team of vascular surgeons and interventional radiologists based on standard clinical guidelines. Target lesions were treated endovascularly with PTA. Stent placement was performed if there was residual stenosis of >30%, flow-limiting dissections, or acute recoil. Target lesions were scored according to the TransAtlantic Inter-Society Consensus for Management of Peripheral Arterial Disease (TASC-II) and the Global Limb Anatomic Staging System (GLASS) by the executing interventional radiologist [3,19]. Procedural details and the entire completion angiography run of the PTA, including images without contrast, were saved in the picture archive and communication system. Revascularization treatment characteristics and arterial segments were grouped for patients with Rutherford classs 2 to 4 and patients with Rutherford class 5.

### 2.3. Angiographic and Clinical Results Post-EVT

Completion angiograms were evaluated by two vascular specialists (J.P.V. and R.B.) blinded to the results of tissue perfusion imaging. A “good” angiographic result of the treated feet was accomplished when a TASC-II C or D lesion was treated and uninterrupted flow continued in at least one below-the-knee artery in continuation with the inframalleolar arteries, resulting in expected increased perfusion post-EVT. If the completion angiogram did not meet these criteria, the angiographic result was classified as “poor,” and tissue perfusion was not expected to increase post-EVT. Angiographic results in which there was disagreement were discussed to reach consensus.

Clinical outcomes were evaluated 6 weeks after revascularization in the outpatient clinic. Clinical improvement was defined as a decrease of at least one Rutherford stage for patients with Rutherford stages 2 to 5. Additionally, for patients with Rutherford stage 5, the wound surface area was clinically evaluated by a wound care nurse together with a vascular specialist and compared with the pre-EVT wound surface area. Improvement was defined when wound surface area decreased or completely healed. Duplex ultrasound imaging was performed pre-EVT and during the first visit to the outpatient clinic at 6 weeks post-EVT according to the standard of care to determine patency of treated arterial lesions. Increased risk for stenosis is defined as a peak systolic velocity of >180 cm/s or a peak velocity ratio across the stenosis of >2.0 [20,21]. Subsequently, the ABI was determined pre-EVT and during the visit to the outpatient clinic at 6 weeks post-EVT.

### 2.4. Perfusion Measurements with HSI and Thermal Imaging

**Figure 1:**
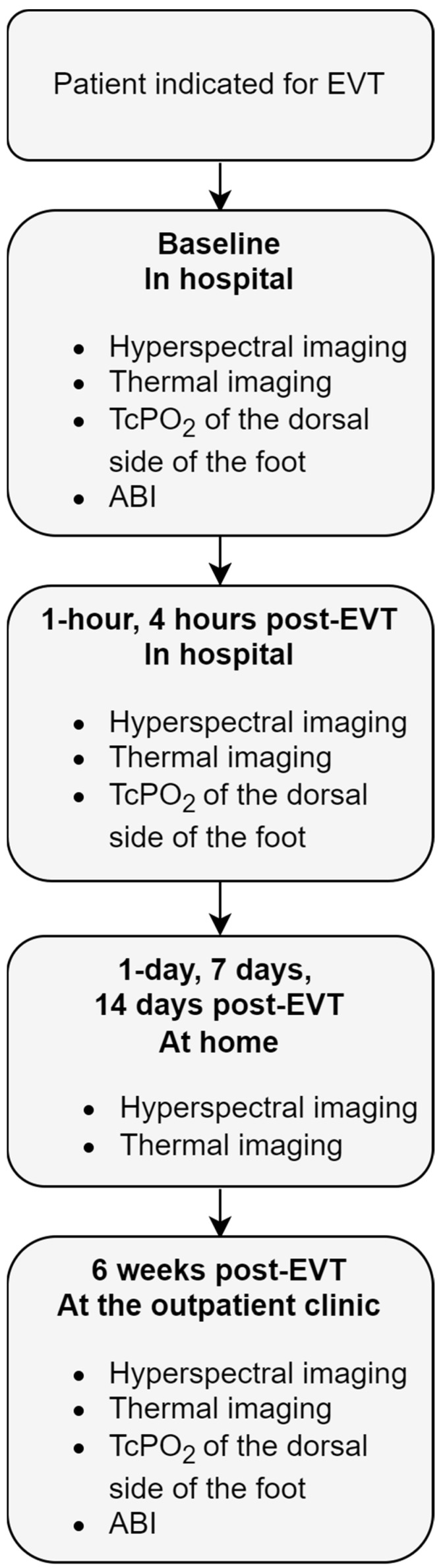
Flowchart of the measurement protocol. ABI, ankle-brachial index; EVT, endovascular therapy; TcPo_2_, transcutaneous oxygen pressure measurement.

HSI is a technique that uses visible light spectroscopy to estimate the concentration of oxyhemoglobin (OxyHb) with 2 absorption peaks at approximately 542 nm and 578 nm and deoxyhemoglobin (DeoxyHb) with a single absorption peak at approximately 554 nm in the upper 1 to 2 mm of the skin [16]. Intra- and inter-observer reliability of this technique is previous described by Kleiss et al. [22] Thermal imaging determines the temperature of the skin with infrared light [23]. HSI was performed with the HyperView system (HyperMed Inc., Memphis, TN, USA). Thermal imaging was performed with a forward-looking infrared (FLIR) camera (FLIR Systems Inc., Wilsonville, OR, USA). Both systems are hand-held cameras that are easy to use and therefore facilitate monitoring patients over time. HSI and thermal imaging were performed pre-EVT at the day of admission, which was considered the baseline measurement for each patient. Six follow-up measurements were performed (Figure 1): 1 h and 4 h post-EVT at the surgical ward, 1 day, 7 days, and 14 days post-EVT at home (unless the patient was in hospital for an appointment), and during the regular scheduled outpatient clinic visit 6 weeks post-EVT.

Patients were in a resting position for at least 5 min before the measurements. Patients were standardly measured in supine position in hospital and at home and in semi-Fowlers position when this was not possible in the home setting. Subsequently, HSI and thermal images of the plantar and dorsal sides of the foot were taken. The image acquisition was standardized for both in-hospital and home measurements, as previously described by Kleiss et al. [16] Home measurements were performed by the researchers of this study (K.M., S.K., or T.N.). The room temperature was standardized in hospital at 21 °C but could not be standardized at home. Conditions that might have influenced the measurements, including ambient light intensity and positioning of legs and feet, were standardized as far as possible for home measurements. Ambient light intensity was standardized by closing the curtains, and patients were placed on a bed, couch, or chair in a semi-Fowler position. During home measurements, patients were assessed regarding PAD; in case of concerns, patients were referred to the outpatient clinic. During in-hospital measurements, transcutaneous oxygen pressure (TcPo_2_) was additionally determined for 15 min using the PeriFlux 6000 TcPo_2_ (Perimed AB, Järfälla, Stockholm, Sweden) on the dorsal side of the foot with a temperature of 44 °C.

### 2.5. Image Preprocessing

**Figure 2:**
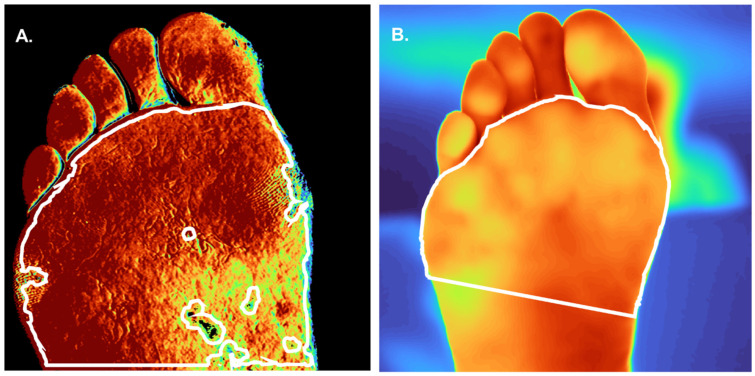
Example of manual segmentations of a thermal and hyperspectral image taken pre-operatively. The patient was a former smoker with Rutherford class 3. (**A**) Hyperspectral image (HSI) of oxyhemoglobin of the plantar side of the foot measured with the HyperView. A darker red color means a higher oxyhemoglobin value. (**B**) Thermal image of the plantar side of the foot measured with the forward-looking infrared (FLIR) camera. More yellow in the color of the foot means a higher temperature value. The white line represents the manual segmentation after preprocessing.

The HyperView device generates an image with the estimated concentration of OxyHb and an image of DeoxyHb. The FLIR camera records a single thermal image. Image postprocessing was performed in Matlab R2018a (Mathworks Inc., Natick, MA, USA). After image acquisition, a region of interest (ROI) was manually drawn on the HSIs, and another ROI was manually drawn in the thermal images at the same spot. For the plantar foot, the ROI was placed at the forefoot as shown in Figure 2. Toes were not included in the ROI, because the surface of the toes is not perpendicular to the HSI camera during image acquisition, which is a requirement for optimal acquisition quality. The segmentation on the dorsal side of the foot included all of the tissue in the image proximal of the caput metatarsal bones. The HSI values at the edges of the foot are not perpendicular to the camera, and therefore, a margin of 3 mm of the edges of the foot were removed before analysis. Artifacts within the ROI were manually excluded from the HSIs, which is shown in Figure 2A. Mean OxyHb value, DeoxyHb values, and temperature inside the ROI were determined for each measurement moment.

### 2.6. Statistical Analysis

Data were recorded using the electronic data management service REDCap (Vanderbilt University, Nashville, TN, USA). Statistical analysis was performed using SPSS 23 software (IBM Corp, Armonk, NY, USA). According to data distribution, descriptive statistics are presented as mean ± standard deviation or median with interquartile range (IQR; 25th and 75th percentile). For each follow-up measurement, the change in perfusion parameters was calculated by subtracting the baseline measurement from the follow-up measurement. Differences between patients with or without clinical improvement or with and without angiographic result for each follow-up moment were calculated using a Mann-Whitney U test. A *p* value of ≤0.05 was considered statistically significant.

## 3. Results

Thirty-four patients participated in the study. There were some missing data of HSI, thermal images, and TcPo_2_ at different time points during follow-up due to logistical reasons such as the lockdown due to the COVID-19 pandemic and inability of patients to visit the hospital for an appointment. Other reasons for missing data were technical failure of the thermal imaging camera during 10 different follow-up assessments and withdrawal of one patient from the study during follow-up. Four measurements were excluded because artifacts were present in >5% of the ROI of the HSI measurements. An overview of the missing data per measurement moment is shown in Supplementary Figure S1.

### 3.1. Patient and Procedural Characteristics

The mean age of the 34 patients was 66.9 ± 9.8 years. Patient characteristics are summarized in Table 1. Seven patients underwent treatment of two limbs simultaneously, which resulted in 41 treated limbs. Table 2 provides an overview of the characteristics of the 41 treated limbs and target lesions (n = 79). The Rutherford classification was five in 37% of the limbs and was Rutherford two to four in 63%. The mean ABI was 0.64 ± 0.20 for the Rutherford class two to four group and 0.61 ± 0.26 for the Rutherford class five group. Ten ABIs were missing due to noncompressible vessels or wounds. Below-the-knee arteries were treated in 18% of the Rutherford class two to four and in 60% of the Rutherford class five.

Postprocedural completion angiograms of the limbs were not available for two treated limbs (5%). Completion angiograms of 13 limbs (32%) showed a good angiographic result, and completion angiograms of 26 limbs (63%) showed a poor angiographic result. Clinical improvement was present in 28 limbs (68%) 6 weeks post-EVT at the outpatient’s clinic visit. From the nine limbs (22%) with Rutherford class two, three (33.3%) had improved clinically, eight (80%) improved from Rutherford class three, and seven (100%) improved from Rutherford class four. Ten (67%) limbs with Rutherford class five improved clinically or showed a decreased wound surface area. Nine (23%) limbs clinically improved and showed a good angiographic result. Nine (23%) limbs had no clinical improvement and had a poor angiographic result. Four (10%) limbs showed a good angiographic result but were not improved clinically. Seventeen (41%) limbs were clinically improved but showed poor angiographic result.

### 3.2. Perfusion Changes Post-EVT in Hospital and at Home

**Figure 3:**
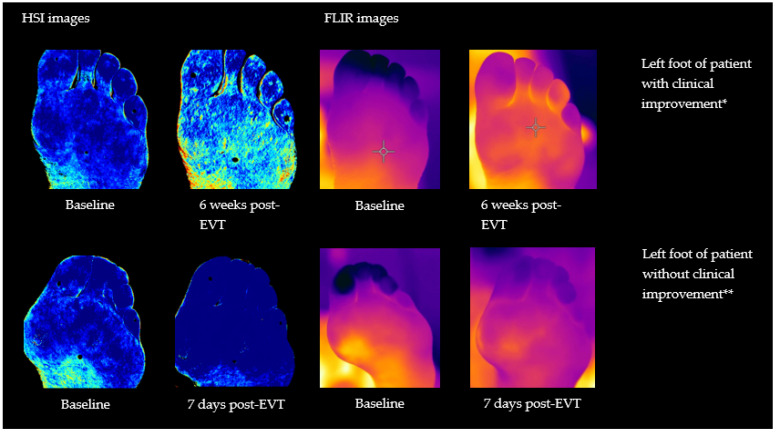
Hyperspectral (HS) images of the deoxyhemoglobin concentration with the HyperView system and FLIR (thermal) images of the plantar side of the left foot of a patient with clinical improvement * at baseline and 6 weeks post-EVT and without clinical improvement ** at baseline and 7 days post-EVT. A darker blue color in the hyperspectral images means a higher deoxyhemoglobin value. A darker purple color means a lower temperature value and more yellow color means a higher temperature value for the thermal images. * Patient does not smoke and has a Rutherford class four. ** Patient was a former smoker with Rutherford class three.

Figure 3 shows an example of hyperspectral images of the deoxyhemoglobin concentration and thermal images of a patient with and without clinical improvement before and after endovascular therapy. Baseline and 6 weeks post-EVT images are displayed for the patient with clinical improvement and baseline and 7 days post-EVT images are displayed for the patient without clinical improvement. The patient with clinical improvement had a Rutherford class of zero at 6 weeks post-EVT, whereby the patient without clinical improvement showed no change in Rutherford class.

**Figure 4:**
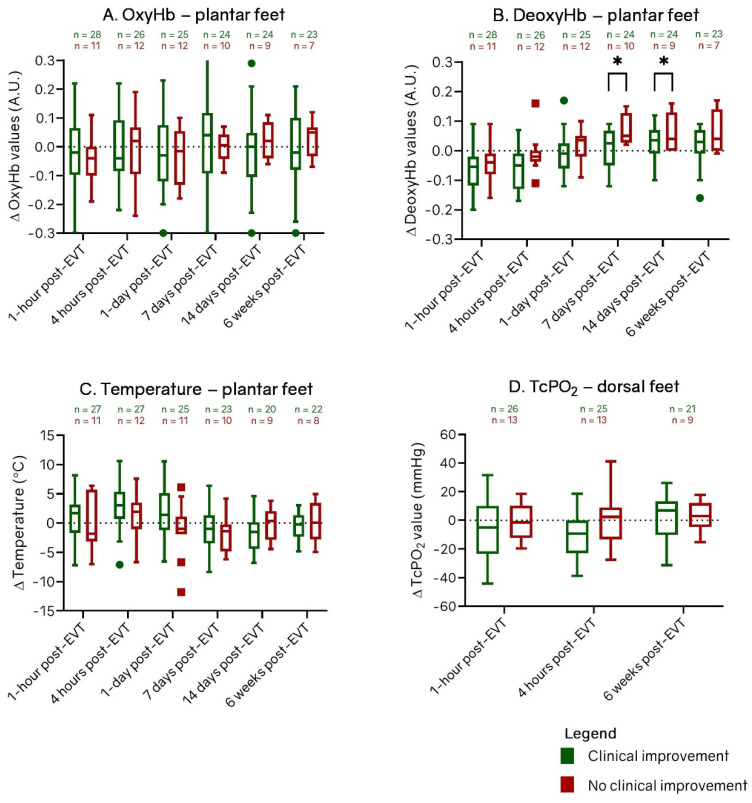
Box-and-whisker plots of change between patients with and without clinical improvement in (**A**) oxyhemoglobin (OxyHb), (**B**) deoxyhemoglobin (DeoxyHb), and (**C**) temperature values at the plantar side and (**D**) change in transcutaneous oxygen pressure (TcPo_2_) values at the dorsal side of all treated feet after endovascular therapy (EVT). Groups are divided in limbs with (green) and without (red) clinical improvement at 6 weeks. The horizontal line in the middle of each box indicates the median; the top and bottom borders of the box mark the 75th and 25th percentiles, respectively, and the whiskers mark the highest value and lowest value within 1.5 × IQR of the 75th and 25th percentiles. The dots are outliers of patients with clinical improvement and the squares are outliers of patients without clinical improvement. A.U.: arbitrary units. * *p* < 0.05.

**Figure 5:**
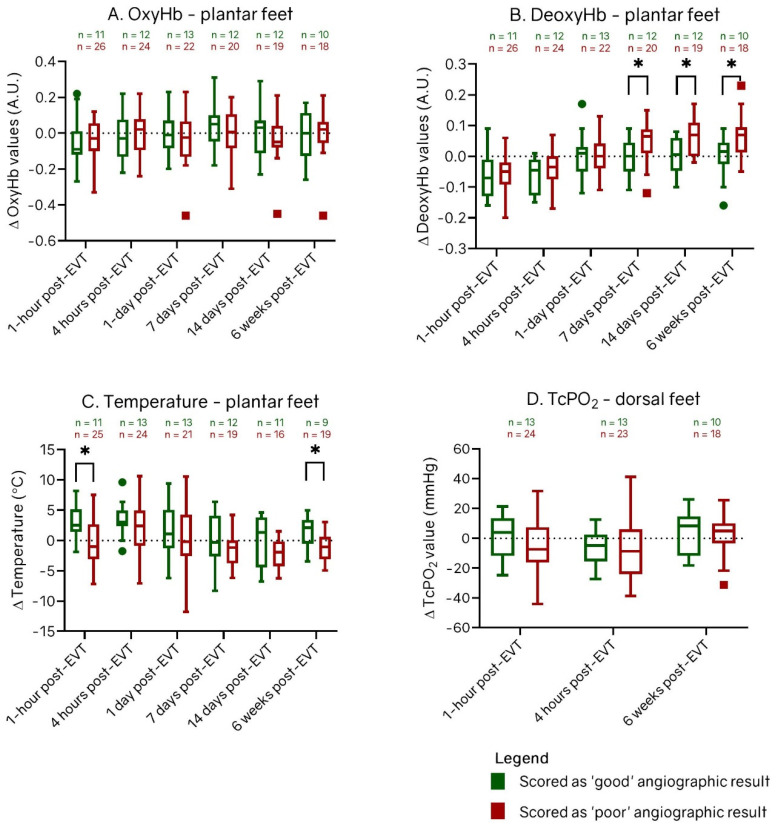
Box-and-whisker plots of change between patients with and without a good angiographic result in (**A**) oxyhemoglobin (OxyHb), (**B**) deoxyhemoglobin (DeoxyHb), (**C**) temperature values at the plantar side and (**D**) change in transcutaneous oxygen pressure (TcPo_2_) values at the dorsal side of all treated feet after endovascular therapy (EVT). Groups are divided in limbs scored as good angiographic result (green) and as poor angiographic result (red). The horizontal line in the middle of each box indicates the median; the top and bottom borders of the box mark the 75th and 25th percentiles, respectively, and the whiskers mark the highest value and lowest value within 1.5 × IQR of the 75th and 25th percentiles. The dots are outliers of patients with clinical improvement and the squares are outliers of patients without clinical improvement. A.U.: arbitrary Units. * *p* < 0.05.

Figure 4 shows the change of OxyHb, DeoxyHb, and temperature per measurement assessment at the plantar side and TcPo_2_ at the dorsal side of all treated limbs post-EVT for patients with and without clinical improvement with respect to baseline measurements. Figure 5 shows the change of OxyHb, DeoxyHb, temperature at the plantar side, and TcPo_2_ at the dorsal side of all treated limbs during follow-up post-EVT for patients with and without good angiographic result with respect to baseline measurements. The perfusion values showed the same trend for all groups. OxyHb values and TcPo_2_ values did not change post-EVT with respect to baseline values. DeoxyHb values were higher directly post-EVT with respect to baseline but decreased over time. Temperature values were higher directly post-EVT with respect to baseline but decreased over time.

Figure 4 shows that the change of DeoxyHb with respect to the baseline values at day 7 was significantly greater in the limbs without clinical improvement than in limbs with clinical improvement (0.08 [IQR, 0.04, 0.15] vs. 0.02 [IQR, −0.05, 0.07]; *p* = 0.009). Similar results were seen at day 14: the limbs without clinical improvement showed a significantly higher increase in DeoxyHb (0.11; IQR, 0.01, 0.15) than limbs with clinical improvement (0.04; IQR, −0.01, 0.07; *p* = 0.049). At all follow-up assessments, there were no statistically significant differences in OxyHb, temperature, or TcPo_2_ between the groups with and without clinical improvement.

Figure 5 shows that the change of DeoxyHb with respect to the baseline values at day 7 was significantly greater in the limbs with poor angiographic result than in limbs with a good angiographic result (0.07 [IQR, 0.01, 0.09] vs. 0.00 [IQR, −0.05, 0.05]; *p* = 0.032). Similar results were seen at day 14: the limbs with poor angiographic result showed a significantly higher increase in DeoxyHb (0.07; IQR, −0.00, 0.11) than limbs with a good angiographic result (0.01; IQR, −0.05, 0.06); *p* = 0.023). Additionally, similar results were determined at 6 weeks post-EVT: the limbs with a poor angiographic result showed a significantly higher increase in DeoxyHb (0.07; IQR, 0.01, 0.09) than limbs with good angiographic result (0.02; IQR, −0.03, 0.04; *p* = 0.045).

The change in temperature with respect to the baseline values at 1 h was significantly greater in the limbs with a good angiographic result than in limbs with a poor angiographic result (2.5° [IQR, 1.4°, 5.1°] vs. −1.0° [IQR, −3.1°, 2.7°]; *p* = 0.018). Similar results were seen at 6 weeks post-EVT: the limbs with a good angiographic result showed a significantly higher increase in temperature (2.1°; IQR, −0.5°, 3.4°) than the limbs with a poor angiographic result (−1.0°; IQR, −3.1°, 0.6°; *p* = 0.025). At all follow-up assessments, there were no statistically significant differences in OxyHb and TcPo_2_ between the groups with a good and poor angiographic result. Differences in OxyHb, DeoxyHb, and temperature of the dorsal side showed the same trend as the perfusion results of the plantar side of the feet. However, no statistical differences were detected between the groups with and without clinical improvement or good angiographic result on this side of the foot.

## 4. Discussion

This prospective study shows the feasibility of tissue perfusion measurements at home post-EVT in patients with PAD. Significant differences in DeoxyHb values at the plantar side of the feet between the groups with and without clinical improvement were detected at 7 days post-EVT and remained up to 6 weeks post-EVT. For the groups with and without a good angiographic result, significant differences in DeoxyHb values were determined at 7 and 14 days. Significant differences in temperature were only found at 1 h and 6 weeks post-EVT between the groups with and without a good angiographic result.

When considering EVT, restoring blood flow may not always instantly translate into detectable perfusion improvement, which is the main goal of treatment. It is, however, of utmost importance to determine whether endovascular revascularization has led to tissue perfusion improvement. Inflammation, ischemic reperfusion injury, and damage to the microvasculature resulting from chronic ischemia can impair actual tissue perfusion during the first hours and days after EVT [13]. The increase in temperature values directly post-EVT and the higher DeoxyHb values in this study can be a result of these factors. It takes 7 days for the microvasculature to regenerate to a steady state, and that is when changes can be detected between patients with and without clinical improvement or a good angiographic result. This implies that restoring the blood flow can lead to improvement of tissue perfusion, but not in patients with a microvasculature that is too greatly affected, which can be captured 7 days post-EVT with HSI in patients with PAD.

Studies investigating the improvement or other changes in perfusion after revascularization are limited [13]. Previous studies reported that improvement in perfusion after EVT was not significantly different in patients with and without diabetes mellitus (DM) but that patients with DM and neuropathy are at highest risk for microcirculatory disfunction [24,25]. The results of other studies suggest that the likelihood of improving flow that may translate to clinical benefit is lower in patients with CLTI compared with patients with intermittent claudication due to the multifactorial and multilevel and complex anatomy of disease in patients with CLTI [26,27]. In our study, clinical improvement at 6 weeks post-EVT was higher in patients with Rutherford stages four to six compared with Rutherford stages two and three (59% vs. 77%).

This study demonstrates that DeoxyHb is the most suitable parameter for tissue perfusion measurements with HSI, which is in accordance with findings in prior studies investigating periprocedural HSI changes at the plantar side of the foot. Oxygenation results (OxyHb andTcPo_2_) did not seem to change, indicating that oxygen extraction (increasing DeoxyHb) seems to increase potentially as a function of deteriorating blood flow over time. Chin et al. [28] showed a reduced DeoxyHb value in patients with PAD compared with patients without PAD, which was not visible in the OxyHb values. Kleiss et al. [18] also demonstrated differences in DeoxHb levels directly after EVT at the plantar side of the feet and showed that DeoxHb was considered acceptable for diagnostic accuracy of short-term clinical improvement.

The current study indicates that follow-up temperature values differ between the patient groups; however, these were not statistically significant at most of the follow-up assessments. Chang et al. [29] investigated whether there was a difference in plantar thermography values between a wound-healing group and a nonhealing group before and after EVT and showed significantly higher temperatures in the healing group. The discrepancy between these findings can be attributed to the heterogeneity of included patients, the number of included patients, or the measurement conditions.

Previous studies have shown that significant differences in TcPo_2_ can be found after ≥6 weeks with respect to baseline values; however, this was not demonstrated within this study. Wagner et al. [30] measured TcPo_2_ before EVT, at 1 day and 6 weeks after EVT in claudicant patients and patients with CLTI and demonstrated significant differences at 6 weeks compared with baseline. Recently, Gunnarsson et al. [31] showed a significant increase in TcPo_2_ 10 weeks after EVT in 21 patients with CLTI.

Home monitoring will become more and more important in this multimorbid, fragile, and immobile vascular diseased patient group, especially in the first weeks post-EVT at home. With an aging population, the call for noninvasive diagnostic techniques that enable the monitoring of tissue perfusion in the home setting can prevent the burden of expensive visits to outpatient clinics and hospitals. Tissue perfusion measurements with HSI could potentially successfully accomplish this; however, it should be integrated within the care protocol of wound consultants or general practitioners. Moreover, a standardized protocol should be developed to overcome variability and user dependency by different care providers. Results of tissue perfusion measurements in the home setting should be integrated within hospital systems to provide the treating physician with valuable information about the patient’s recovery, which could decrease the need for hospital visits and improve early detection of the need for additional (re)interventions for patients with PAD.

### Limitations

This study is the first step toward noninvasive, contact-free home monitoring with HSI, although there are some limitations. One of the study limitations is the small group of 34 patients and a substantial number of missing measurements. Fewer PTA procedures were scheduled because of COVID-19, and patients were less willing to participate within the study. Home monitoring was not permitted during the COVID-19 lockdown, which resulted in missing the data of home measurements. A major downside of all the missing data was the inability to perform mixed-model statistical analyses, which was the preferred method within longitudinal data analyses and should be used within future studies.

When considering the homogeneity of the groups in this study, it should be noted that the classification of the groups in our study was based on subjective measures and did not include objective measures such as ankle pressure, toe pressure, ischemia, or infection status of the wounds. This study included eight patients with DM in the Rutherford two to four group and seven patients with DM in the Rutherford five and six group, which disturbs the homogeneity of the groups. This may be the reason that no differences were found in ABIs between the groups with Rutherford two to four and Rutherford five and six and that TcPo_2_ showed no significant differences between the groups with and without clinical improvement or good angiographic result.

Several other factors may affect tissue perfusion measurements, which were previously described by Kleiss et al. [22]. These factors, such as physical activity, stress, smoking, caffeine intake, differences in environmental temperature, and ambient light intensity, were difficult to standardize during measurements in this study, especially in the home setting. For this reason, the intensity in the HSI and thermal images varied a lot throughout the time. Physical activity was not measured in this study. The temperature of 21 °C in hospital should be considered as a factor that influenced the absolute perfusion values, but was standardized. It was not possible to standardize the room temperature within the home setting. The reason for a decreasing trend, seen in the temperature of the feet over time, could be a result of the decrease in vascular health. However, this is not certain due to interfering factors.

Finally, the ROI of the HSIs was not automatically translated to the thermography images, and artifacts were manually removed of the images. This manual segmentation of HSI and thermal images could have introduced variability in the area that is segmented, which is intra-observer and inter-observer dependent. For future studies, segmentation based on intensity or machine learning should be investigated.

## 5. Conclusions

This prospective pilot study shows the feasibility of HSI and thermal imaging post-EVT at home, which may decrease the need for hospital visits. Significant differences in DeoxyHb levels at 7 and 14 days post-EVT were determined between patients with and without clinical or angiographic success.

## Figures and Tables

**Table 1:** Patient characteristics.

Variables	Data Value
	(N = 34)
Age, years	66.9 ± 9.8
Sex	
Male	24 (71)
Female	10 (29)
Body mass index, kg/m^2^	26.0 ± 4.3
Smoking	
Current	12 (35)
Former smoker *	17 (50)
Nonsmoker	5 (15)
Hyperlipidemia	8 (24)
Diabetes mellitus	15 (44)
Type 1	1 (3)
Type 2	14 (41)
Hypertension	19 (56)
Coronary artery disease **	21 (62)
Prior cerebral events	8 (24)
Pulmonary disease ***	13 (38)
Renal dysfunction (eGFR < 60 mL/min/1.73 m^2^)	8 (24)

Data are presented as mean ± standard deviation or number (%). eGFR, estimated glomerular filtration rate. * Patients who have smoked in the past but have stopped > 1 month before inclusion. ** Myocardial infarct, percutaneous coronary intervention, or coronary artery bypass grafting. *** Chronic obstructive pulmonary disease, asthma, or emphysema.

**Table 2:** Revascularization treatment characteristics of feet (n = 41) and arterial segments (n = 79).

Variable	Rutherford Class 2 to 4	Rutherford Class 5
	(N = 26)	(N = 15)
Rutherford classification		
2	9 (35)	-
3	10 (38)	-
4	7 (27)	-
5	-	15 (100)
ABI (N = 31)	0.64 ± 0.20	0.61 ± 0.26
GLASS classification		
Infrainguinal GLASS stage		
N/A	18 (69)	5 (33)
I	1 (4)	4 (27)
II	1 (4)	4 (27)
III	6 (23)	2 (13)
Not classified ^a^	-	-
Inframalleolar	1 (4)	0 (0)
P0	12 (46)	6 (40)
P1	3 (12)	1 (7)
P2	6 (23)	7 (47)
Not classified ^a^	4 (15)	1 (7)
Treated arterial segments	(N = 44)	(N = 35)
Arterial segments		
CIA	11 (25)	3 (9)
EIA	13 (30)	3 (9)
SFA	12 (27)	8 (23)
PFA	0 (0)	0 (0)
Popliteal artery	4 (9)	8 (23)
ATA	2 (5)	7 (20)
Peroneal artery	2 (5)	4 (11)
Posterior tibial artery	0 (0)	2 (6)
TASC-II classification		
TASC-II A	18 (41)	13 (37)
TASC-II B	11 (25)	12 (34)
TASC-II C	6 (14)	6 (17)
TASC-II D	9 (20)	4 (11)
Endovascular therapy		
PTA without stent	10 (23)	21 (60)
PTA and stent	34 (77)	14 (40)

Data are presented as mean ± standard deviation or number (%). ABI, ankle-brachial index; ATA, anterior tibial artery; CIA, common iliac artery; EIA, external iliac artery; GLASS, Global Limb Anatomic Staging System; PFA, deep femoral artery; PTA, percutaneous transluminal angioplasty; SFA, superior femoral artery; TASC-II, TransAtlantic Inter-Society Consensus for Management of Peripheral Arterial Disease-II. ^a^ Not classified due to insufficient imaging quality.

## Data Availability

The data presented in this study are available on request from the corresponding author. The data are not publicly available due to privacy and ethical reasons.

## Supplementary Materials

The following supporting information can be downloaded at: https://www.mdpi.com/article/10.3390/diagnostics12102489/s1, Figure S1: Flowchart of missing data at baseline, 1 h post-endovascular therapy (EVT), 4 h post-EVT, 1-day post-EVT, 7 days post-EVT, 14 days post-EVT, and 6 weeks post-EVT for hyperspectral imaging (HSI), thermal imaging, and transcutaneous oxygen pressure (TcPO2) measurements. *Missed due to coronavirus disease 19 (COVID-19) or inability of patient to visit the appointment. The HyperView device was used for hyperspectral imaging. The forward-looking infrared (FLIR) camera was used for temperature measurements.
