# Supplementary material for: Determination of Changes in Tissue Perfusion at Home with Hyperspectral and Thermal Imaging in the First Six Weeks after Endovascular Therapy in Patients with Peripheral Arterial Disease
Source: Diagnostics (Basel). 2022 Oct 14;12(10):2489. doi: 10.3390/diagnostics12102489 (PMC9600062; doi:10.3390/diagnostics12102489)
Supplement: Supplementary file 1 [file diagnostics-12-02489-s001.zip › diagnostics-1933551-supplementary.pdf]

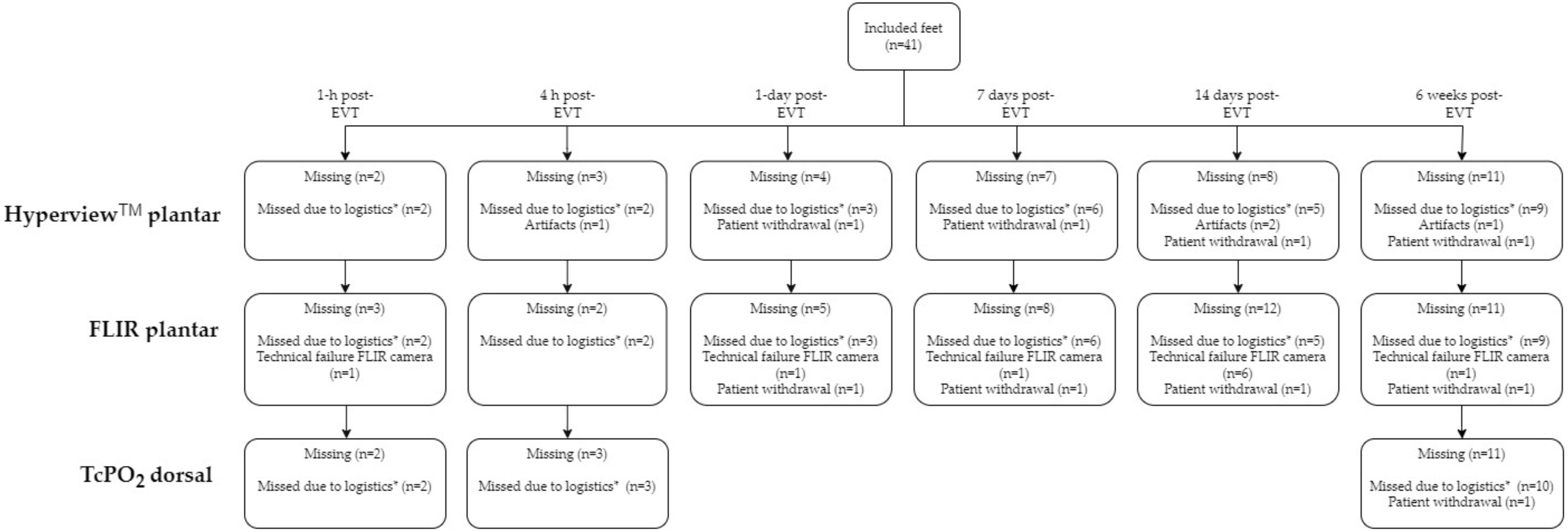

Figure S1. Flowchart of missing data at baseline, 1 h post-endovascular therapy (EVT), 4 h post-EVT, 1-day post-EVT, 7 days post-EVT, 14 days post-EVT, and 6 weeks post-EVT for hyperspectral imaging (HSI), thermal imaging, and transcutaneous oxygen pressure (TcPO2) measurements. *Missed due to coronavirus disease 19 (COVID-19) or inability of patient to visit the appointment. The HyperView device was used for hyperspectral imaging. The forward-looking infrared (FLIR) camera was used for temperature measurements.
